# Supplementary material for: Rapid Differentiation between Microplastic Particles Using Integrated Microwave Cytometry with 3D Electrodes
Source: ACS Sens. 2025 Mar 18;10(3):1729–35. doi: 10.1021/acssensors.4c03268 (PMC11959584; doi:10.1021/acssensors.4c03268)
Supplement: Supplementary file 1 — se4c03268_si_001.pdf [file se4c03268_si_001.pdf]

Supplementary Information for

Rapid Differentiation between Microplastic Particles Using Integrated Microwave Cytometry with 3D Electrodes

Yagmur Ceren Alatas[1,2], Uzay Tefek[1,2], Sayedus Salehin[1,2], Hashim Alhmoud[1,2] and M. Selim Hanay[1,2,*]

1 Department of Mechanical Engineering, Bilkent University,06800 Ankara, TURKEY

2 UNAM – Institute of Materials Science and Nanotechnology, Bilkent University, 06800, Ankara TURKEY

This PDF includes:

8 pages, 4 Figures, 1 Table

Component List for the Measurement Circuitry

Two lock-in amplifiers (Zurich Instruments, MFLI at the top; 1x Zurich Instruments, HF2LI at the bottom of the Figure 3b)

Transconductance amplifier (Zurich Instruments, HF2TA)

Signal generator (Rohde & Schwarz SMB-100A)

Power splitter (Mini-Circuits, ZN2PD2-14W-S+)

IQ mixer (Marki Microwave, MMIQ0218LXPC)

Circulator (Pasternack, PE8402)

Mixer (Mini-Circuits, ZX06-U742MH-S)

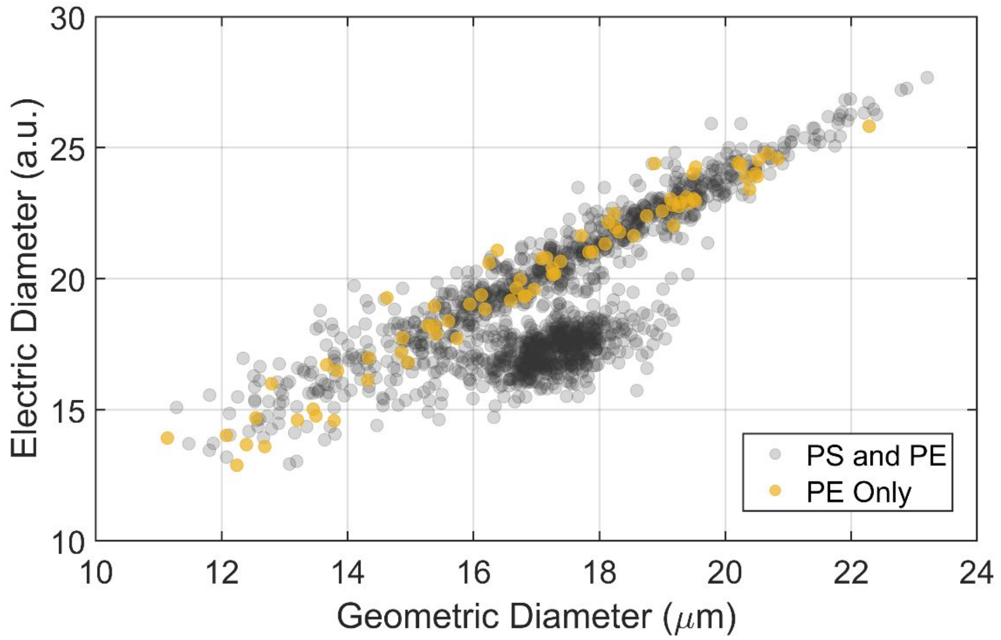

Figure S1. Polyethylene only measurement plotted with mixed particles measurement results. The distribution range of polyethylene is used to calibrate the PE-only data.

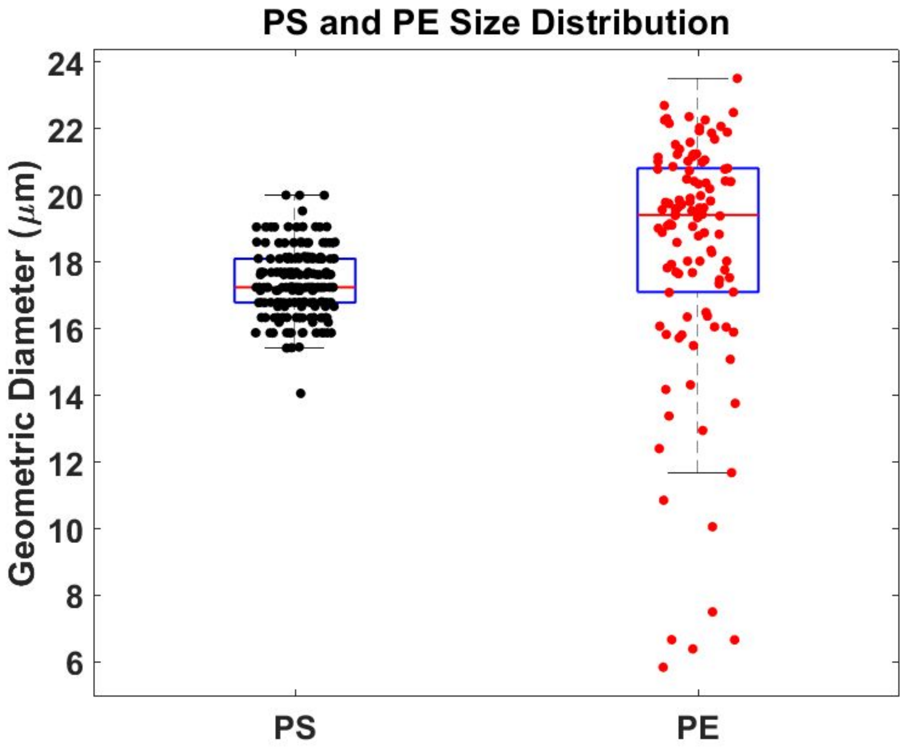

Figure S2. Size distribution of 200 Polystyrene (PS) and 110 Polyethylene (PE) microparticles from the images taken from high resoulution optical microscopy.

According to the vendor's specificaitons the diameter range of polystyrene is 14-20 µm and the diameter range of polyethylene is 10-20 µm. However, according to the microscopy images of the particles which alignes much better with the experimental results, the mean diameter of polystyrene is 17.24 (SD:0.97) µm and the mean diameter of polyethylene is 19.21 (SD:3.7) µm.

For calibration, we use the narrow size range of the Polystyrene (PS): in each run where there are PS particles, their mean size was adjusted to match the mean size of the microscopy value (17.24 µm). This normalization of geometry also normalizes the PE, and their size range comes out to be match the 10-24 µm size range, as expected. There is only run without the PS particles available for calibration: the PE-only run. In this case, the size range was normalized to the known size range of the PE particles. With this geometric normalization, the PE-only data overlaps with the PE-region of the PS-PE mixture data.

Multiple Events

Shown below are two waveforms: in the first one it is clear that a single particle has passed; but on the second one, two particles passed back-by-back, effecting each others' waveform. While it is plausible that the waveforms could be disentangled, we opted to eliminate such occurrence for simplicity of the analysis, and the fact that microplastic samples are dilute. 94% of the events were deemed valid, and 6% of the events were disregarded on the basis that two particles passed through the active region within a short duration.

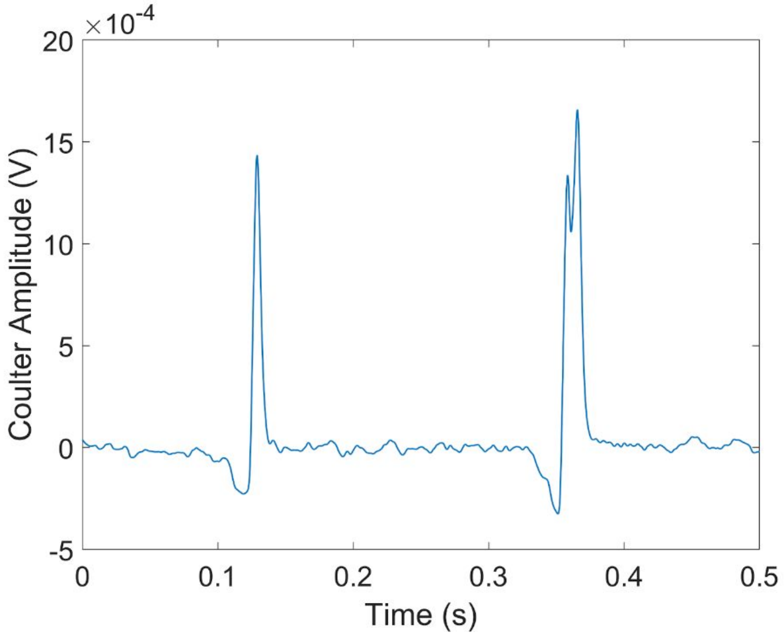

Figure S3. The left one is a single event (valid), the right one is a multi-particle event (invalid)

Dielectric Constant Models / Circuit Model

Below is the comparative analysis of 11 different modeling equations used to characterize the microwave sensor's sensing region. The sensing region is defined as the volume between the electrodes (50 μm height, 30 μm width, and 50 μm apart), where capacitance changes caused by microparticles are measured.

The y-axis represents the ratio of the capacitance change for polystyrene to polyethylene for a given volume ratio of the particles to the sensing region. The x-axis denotes the equation numbers: Parallel (1), Series (2), Logarithmic (3), Lichtenecker (4), Looyenga (5), Birchak (6), Poon-Shin (7), Electro-Magnetic Theory (EMT) (8), Maxwell-Garnett (9), Jayasundere–Smith (10), and a combination of Parallel and Series (11). Each color in the plot corresponds to a specific volume ratio, ranging from 0.02 to 0.1.

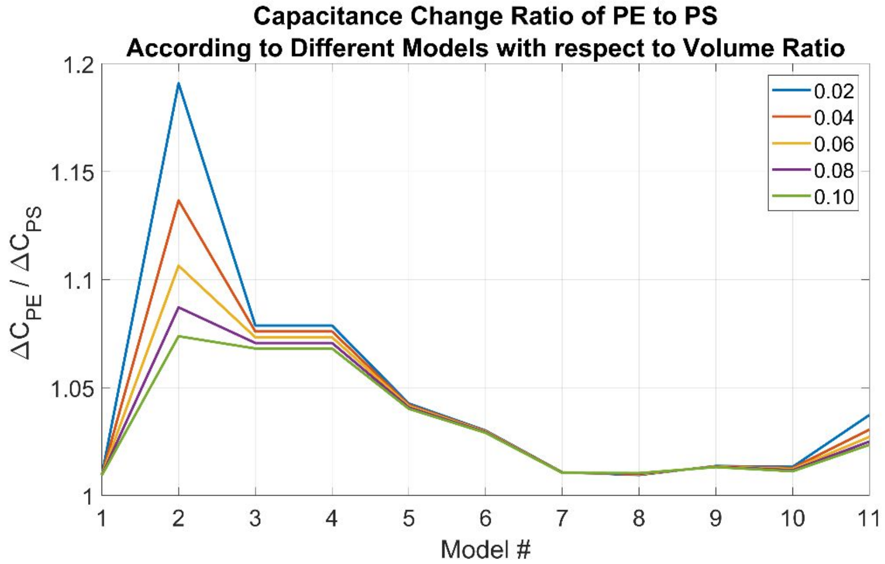

Figure S4. The ratio of the capacitance change caused by polystyrene to polyethylene particles for different volume ratios.

The experimental results indicate that the ratio of the capacitance change caused by polystyrene to polyethylene particles is 1.18, significantly higher than the ratios predicted by most models, which cluster around 1.02. Among the models considered, only the Series (2nd) model closely matches the experimentally observed value across all volume ratios (V_p:particle volume ratio, V_m:medium volume ratio) suggesting that it more accurately captures the physical interactions in the sensing region. For all the models, the following parameters are used:

$$V_p = 0.04,\ V_m = 0.96,\ \varepsilon_{p,ps} = 3,\ \varepsilon_{p,pe} = 2.3,\ \varepsilon_{m,} = 78,$$

In order to calculate an approximate V_p, the mean size of polystyrene particles (radius = 9 μm) and sensing region dimensions are used. With the given parameters, the Series model yields a capacitance change ratio of 1.14 for polystyrene and polyethylene particles.

The discrepancy between the experimental results and the predictions of most models likely arises from differences in how the models handle several factors. Microscale effects such as surface charge, conductivity, roughness, and compositional non-uniformity can also affect the dielectric properties. By its nature, the Series model might better account for such interfacial phenomena. Non-uniformities in particle packing may influence the capacitance response, and the series model might better approximate this effect. Moreover, many models assume ideal dielectric

behavior for the particles, while the experimental setup involves complex interactions not fully captured by such models.

Models:

$$\varepsilon_{mix} = V_p\varepsilon_p + V_m\varepsilon_m (S1)$$

$$(\varepsilon_{mix})^{-1} = V_p(\varepsilon_p)^{-1} + V_m(\varepsilon_m)^{-1} (S2)$$

$$\varepsilon_{mix} = {\varepsilon_p}^{V_p} + {\varepsilon_m}^{V_m} (S3)$$

$$\varepsilon_{mix} = {\varepsilon_p}^{V_p}\, {\varepsilon_m}^{V_m} (S4)$$

$$\varepsilon_{mix}^{\frac{1}{3}} = V_m\varepsilon_m^{\frac{1}{3}} + V_p\varepsilon_p^{\frac{1}{3}} \quad (S5)$$

$$\varepsilon_{mix}^{\frac{1}{2}} = V_m\varepsilon_m^{\frac{1}{2}} + V_p\varepsilon_p^{\frac{1}{2}} (S6)$$

$$\varepsilon_{mix} = \varepsilon_m\left[1 + \frac{V_p\left(\frac{\varepsilon_p}{\varepsilon_m} - 1\right)}{V_p + \left(\frac{V_m}{3}\right)\left(\frac{\varepsilon_p}{\varepsilon_m}V_m + V_p + 2\right)}\right] (S7)$$

$$\varepsilon_{mix} = \varepsilon_m\left[1 + \frac{V_p(\varepsilon_p - \varepsilon_m)}{\varepsilon_m + nV_p(\varepsilon_p - \varepsilon_m)}\right] (S8)$$

$$\varepsilon_{mix} = \varepsilon_m + 3 * V_p\varepsilon_m\left[\frac{\varepsilon_p - \varepsilon_m}{\varepsilon_p + 2\varepsilon_m - V_p(\varepsilon_p - \varepsilon_m)}\right] (S9)$$

$$\varepsilon_{mix} = \frac{\varepsilon_m V_m + \varepsilon_p V_p\left[\frac{3\varepsilon_m}{\varepsilon_p + 2\varepsilon_m}\right]\left[1 + \frac{\left(3V_p(\varepsilon_p - \varepsilon_m)\right)}{\left(\varepsilon_p + 2\varepsilon_m\right)}\right]}{V_m + \frac{3V_p\varepsilon_m}{\left(\varepsilon_p + 2\varepsilon_m\right)}\left[1 + \frac{\left(3V_p(\varepsilon_p - \varepsilon_m)\right)}{\left(\varepsilon_p + 2\varepsilon_m\right)}\right]} (S10)$$

Statistical Comparison of 2D to 3D Electrode Geometries

Table S1:Statistical comparison of 3D to 2D electrode design

	CV of **3D** Electrodes Raw	CV of **2D** Electrodes Raw	CV of **2D** Electrodes Corrected
Geometrical Diameter	0.038	0.023	0.021
Electrical Diameter	0.053	0.048	0.017

Table 1 compares the coefficient of variation (CV) parameters for measurements conducted using two versions of the sensor: 2D and 3D electrode configurations. The 2D sensor was tested with 20 ± 0.3 µm polystyrene particles (since the data came from an earlier paper[1]) while the 3D sensor was evaluated using 30 ± 0.4 µm polystyrene particles. On the last column of the table, the height-corrected values for the 2D sensor is reported.

The 2D electrode configuration features an electrode spacing of 15 µm, which enhances its sensitivity for particles within this size range. As a result, the 2D sensor exhibits superior CV values for particles close to 20 µm. However, measurements for particles significantly larger or smaller than this range show increased error margins. Additionally, the accuracy of the 2D sensor is influenced by particle trajectory, necessitating a height calibration process to achieve optimal resolution.

In contrast, the 3D electrode configuration, with electrodes spaced 50 µm apart, can accommodate a wider range of particle sizes without requiring further height calibration. However, this comes at the cost of reduced resolution compared to the 2D configuration.

References

1. Tefek, U.; Sari, B.; Alhmoud, H. Z.; Hanay, M. S., Permittivity-Based Microparticle Classification by the Integration of Impedance Cytometry and Microwave Resonators. *Advanced Materials* **2023,** *35* (46), 2304072.
